# Altered Monocyte Subsets in Patients with Chronic Idiopathic Neutropenia

**DOI:** 10.1007/s10875-019-00694-5

**Published:** 2019-10-27

**Authors:** Nikoleta Bizymi, Maria Velegraki, Athina Damianaki, Helen Koutala, Helen A. Papadaki

**Affiliations:** 1 Hemopoiesis Research Laboratory, School of Medicine, University of Crete, Heraklion, Greece; 2 Department of Hematology, University Hospital of Heraklion, P.O. Box 1352, Heraklion, Crete, Greece

To the editor,

Chronic idiopathic neutropenia (CIN) of adults is a neutrophil disorder characterized by the persistent and unexplained reduction in the number of peripheral blood (PB) neutrophils below the lower limit of the normal range in a given ethnic population, for a prolonged period (more than 3 months) [1]. The diagnosis of CIN is based on exclusion criteria, namely the absence of clinical and laboratory evidence of any underlying disease that might be associated with neutropenia, absence of history of exposure to irradiation, absence of use of chemical compounds or intake of drugs that might cause neutropenia, negative antineutrophil antibody testing to exclude antibody-mediated immune neutropenia, and normal bone marrow (BM) morphology and karyotype to exclude cases of myelodysplastic syndrome (MDS) presenting as isolated neutropenia [1]. The inclusion in the diagnostic algorithm of a detailed flow cytometric analysis of the BM progenitor cells and next generation sequencing of genes related to myeloid malignancies largely contributes to the recognition of pre-MDS neutropenic patients [1, 2].

The etiology of CIN is not entirely known. However, there is evidence suggesting that the pathophysiology of CIN is related to an inflammatory BM microenvironment consisting of proinflammatory cytokines such as tumor necrosis factor-α, interferon-γ and interleukin-1β, proapoptotic mediators such as Fas-ligand, and activated oligoclonal or monoclonal T lymphocytes with myelosuppressive properties that collectively induce the accelerated apoptotic death of the granulocytic progenitor cells [3]. Increased levels of proinflammatory cytokines are also found in the PB of CIN patients [4]. The cellular origin of these cytokines in the BM and PB of CIN patients remains largely unknown.

The possible involvement of the monocytic lineage in the pathophysiology of CIN has not been studied so far. Normally, circulating monocytes consist of phenotypically and functionally heterogeneous subpopulations, namely the classical (CD14^bright^/CD16^−^) monocytes which represent the majority of PB monocytes displaying mainly phagocytic and tissue repair capacity, the intermediate (CD14^bright^/CD16^+^) monocytes producing inflammatory cytokines in response to inflammatory stimuli, and the non-classical (CD14^dim^/CD16^+^) monocytes displaying proinflammatory properties in association with patrolling and antimicrobial functions [5]. Increased number of CD16^+^ (intermediate and non-classical) monocytes have been reported in infectious and inflammatory conditions such as cardiovascular, chronic kidney, and autoimmune diseases [5].

In the present study, we sought to evaluate by flow cytometry the quantitative characteristics of PB monocyte subsets in CIN patients (*n* = 70) compared to age- and sex-matched healthy individuals (*n* = 22). All patients fulfilled the above-described diagnostic criteria of CIN and had mean neutrophil counts 1127 ± 529/μL (range 100–1700/μL) for a mean period of 144 ± 78 months (range 24–312 months). For the definition of neutropenia, we used the threshold of 1800/μL for the absolute neutrophil count according to the World Health Organization [6]. None of the patients had evidence of active infection, cardiovascular, chronic kidney, or autoimmune disease. Detailed patient characteristics are shown in the Supplemental Table 1. The study has been approved by the Ethics Committee of the University Hospital of Heraklion, and informed consent according to the Helsinki Protocol was obtained from all subjects studied. The proportion of the PB monocyte subsets, namely the classical CD14^bright^/CD16^−^, intermediate CD14^bright^/CD16^+^, and non-classical CD14^dim^/CD16^+^ cells, was evaluated on the basis of their relative CD14 and CD16 surface expression using fluorescein isothiocyanate (FITC)-conjugated mouse antihuman CD16 (clone 3G8) and phycoerythrin (PE)-conjugated anti-CD14 (clone RMO52) monoclonal antibodies or the isotypic controls (all purchased from Beckman Coulter, Marseille, France) following red blood lysis (ImmunoPrep reagent system; Beckman Coulter). Analysis was performed in a Cytomics FC500 flow cytometer (Beckman Coulter, Brea, CA, USA) in the gate of the CD14^+^ cells representing the pure monocytic population (Fig. 1a). Data were analyzed by means of the non-parametric Mann-Whitney *t* test and the Spearman correlation test (GraphPad Software, San Diego, CA, USA). Grouped data are expressed as mean ± 1 standard deviation.

**Fig. 1:**
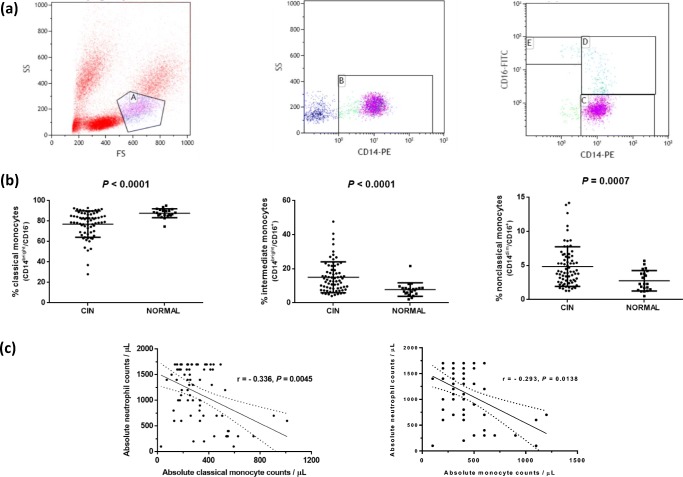
Flow cytometric analysis of peripheral blood monocyte subsets in CIN patients and normal controls and correlations with absolute neutrophil counts. **a** The graphs show an example of flow cytometric analysis of peripheral blood monocyte subsets, namely the classical CD14^bright^/CD16^−^, intermediate CD14^bright^/CD16^+^, and non-classical CD14^dim^/CD16^+^ cells from a healthy individual. The first graph depicts the initial gating of monocytes as cells with intermediate forward scatter (FSC) and side scatter (SSC) properties (gate A). The second graph shows the pure monocyte population (gate B) representing the CD14^+^ cells within gate A. This gating strategy excludes any natural killer cells or neutrophils which also express surface CD16 but devoid of the CD14 antigen. The third graph depicts a representative dot plot of the classical (gate C), intermediate (gate D), and non-classical (gate G) monocytes in the gate of CD14^+^ cells. **b** The graphs show the individual proportions of the classical (CD14^bright^/CD16^−^), intermediate (CD14^bright^/CD16^+^), and non-classical (CD14^dim^/CD16^+^) monocytes detected by flow cytometric analysis within the CD14^+^ cell compartment of CIN patients (circles) and normal subjects (squares). The horizontal lines indicate the mean proportion ± 1 standard deviation of the classical, intermediate, and non-classical monocytes in the groups of CIN patients and controls. Analysis between CIN patients and controls has been performed by using the non-parametric Mann-Whitney *t* test, and the *P* values are indicated. **c** The graphs show the inverse correlation between the absolute neutrophil and the absolute monocyte counts in CIN patients. The first graph shows the inverse correlation between the absolute neutrophil and the absolute classical monocyte counts, and the second graph the correlation between the absolute neutrophil and the absolute total monocyte counts in the patients. The dots represent individual cell counts of CIN patients (*n* = 70), and the solid and dotted lines show the regression line and the 95% confidence limits, respectively, following analysis by means of the non-parametric Spearman correlation test. The coefficient of correlation (*r*) and degree of significance (*P*) are indicated.

Consistent with our previous observations, CIN patients displayed lower PB absolute monocyte counts (419 ± 215/μL) compared to healthy individuals (476 ± 122/μL; *P* = 0.0138) corroborating further the hypothesis for a BM defect in CIN [4]. Indeed, we have previously shown, using BM clonogenic progenitor cell assays, that the BM mononuclear cell fraction of CIN patients consists of lower number of granulocyte-monocyte colony forming units (GM-CFU) compared to healthy controls [3].

As regards to the PB monocyte subsets, we found decreased proportion of classical CD14^bright^/CD16^−^ cells in CIN patients (77.05% ± 12.88%) compared to the controls (87.68% ± 4.38%; *P* < 0.0001). On the contrary, a significant increase was observed in the proportion of the intermediate CD14^bright^/CD16^+^ and the non-classical CD14^dim^/CD16^+^ monocyte subsets in CIN patients (15.03% ± 9.04% and 4.84% ± 2.90%, respectively) compared to the controls (7.80% ± 3.99% and 2.76% ± 1.50%, respectively; *P* < 0.0001 and *P* = 0.0007, respectively) (Fig. 1b). We then calculated the absolute numbers of the above monocyte subsets by multiplying the percentage of each cell subpopulation obtained by flow cytometric analysis with the absolute number of monocytes in the complete blood counts at the time of sampling. A statistically significant decrease was observed in the absolute number of the classical CD14^bright^/CD16^−^ monocytes in CIN patients (328 ± 182/μL) compared to the controls (418 ± 114/μL; *P* = 0.0015) that was associated with a significant increase in the absolute number of the intermediate CD14^bright^/CD16^+^ and the non-classical CD14^dim^/CD16^+^ monocytes in the patients (60 ± 50/μL and 18 ± 12/μL, respectively) compared to the controls (37 ± 19 and 13 ± 8, respectively; *P* = 0.0272 and *P* = 0.0293, respectively). An inverse correlation was found between the absolute neutrophil and the absolute classical monocyte counts in CIN patients (*r* = − 0.336, *P* = 0.0045) indicating possibly a compensatory effort of the BM in response to neutropenia (Fig. 1c). Given that the classical monocytes are the prominent cell subsets among total monocytes, an inverse correlation was also found between patients’ absolute neutrophil and absolute total monocyte counts (*r* = − 0.293, *P* = 0.0138) (Fig. 1c). No correlation was identified between the absolute neutrophil and the intermediate or the non-classical monocyte counts suggesting that any involvement of these cell populations in the pathophysiology of CIN represents a contributory but not the main mechanism of neutropenia.

In conclusion, we show here for the first time that the monocyte cell subsets are altered in CIN patients and consist of decreased number of classical monocytes and increased intermediate and non-classical ones. The intermediate and non-classical monocytes display proinflammatory properties in terms of higher expression of proinflammatory cytokines and higher potential for antigen presentation [5]. We may thus hypothesize that the intermediate and non-classical monocyte subsets contribute to the aberrant proinflammatory cytokine production and T cell activation previously described in CIN. This hypothesis is currently under investigation by profiling the transcriptome of isolated monocyte subpopulations from CIN patients and normal controls. Furthermore, given that the intermediate and non-classical monocytes can migrate into inflammatory tissues through specific chemokine-ligand interactions, we are currently investigating the presence of these cell populations in CIN BM and their contribution in the inflammatory BM microenvironment associated with CIN.

## Electronic supplementary material

Supplemental Table 1 (DOC 121 kb)
