# Supplementary material for: Altered Monocyte Subsets in Patients with Chronic Idiopathic Neutropenia
Source: J Clin Immunol. 2019 Oct 27;39(8):852–4. doi: 10.1007/s10875-019-00694-5 (PMC6863791; doi:10.1007/s10875-019-00694-5)
Supplement: Supplementary file 1 — (DOC 121 kb) [file 10875_2019_694_MOESM1_ESM.doc]

**Supplemental Table 1.** Clinical and laboratory data of the CIN patients studied.

| **UPN** | **Age (years)** | **Sex** | **Duration (months)** | **Hb (g/dl)** | **WBC (x10^6^/L)** | **Neutro (x10^6^/L)** | **Lympho (x10^6^/L)** | **Mono (x10^6^/L)** | **Plts (x10^9^/L)** |
| --- | --- | --- | --- | --- | --- | --- | --- | --- | --- |
| 1 | 35 | F | 120 | 12.7 | 2600 | 300 | 1500 | 500 | 282 |
| 2 | 50 | F | 156 | 13.0 | 4100 | 1200 | 2200 | 600 | 229 |
| 3 | 54 | F | 156 | 13.2 | 2500 | 200 | 1700 | 400 | 225 |
| 4 | 19 | F | 168 | 12.1 | 3200 | 700 | 1800 | 600 | 322 |
| 5 | 27 | M | 228 | 12.5 | 2900 | 700 | 1700 | 300 | 331 |
| 6 | 39 | M | 144 | 13.8 | 2400 | 300 | 1200 | 700 | 150 |
| 7 | 80 | F | 132 | 12.9 | 3500 | 1300 | 1700 | 500 | 112 |
| 8 | 20 | M | 252 | 15.3 | 2800 | 300 | 1700 | 900 | 187 |
| 9 | 57 | F | 252 | 12.6 | 3600 | 1300 | 1600 | 400 | 167 |
| 10 | 48 | F | 228 | 12.3 | 3600 | 1700 | 1400 | 500 | 297 |
| 11 | 38 | F | 60 | 12.4 | 3600 | 1700 | 1300 | 400 | 266 |
| 12 | 67 | F | 252 | 11.2 | 4700 | 1000 | 3100 | 400 | 215 |
| 13 | 53 | F | 60 | 15.1 | 3500 | 1300 | 1900 | 300 | 161 |
| 14 | 56 | F | 60 | 12.8 | 4300 | 1100 | 2600 | 400 | 255 |
| 15 | 61 | F | 156 | 12.2 | 3500 | 1700 | 1500 | 300 | 202 |
| 16 | 58 | M | 120 | 15.8 | 2100 | 600 | 1200 | 200 | 162 |
| 17 | 66 | M | 72 | 14.3 | 4100 | 1700 | 1800 | 300 | 180 |
| 18 | 44 | M | 60 | 12.8 | 4500 | 1700 | 1900 | 600 | 174 |
| 19 | 49 | F | 264 | 12.7 | 3800 | 1100 | 2000 | 300 | 219 |
| 20 | 67 | F | 180 | 12.9 | 3900 | 1700 | 1600 | 300 | 189 |
| 21 | 48 | F | 264 | 14.4 | 3600 | 1700 | 1500 | 400 | 212 |
| 22 | 32 | F | 204 | 12.1 | 2100 | 800 | 1100 | 200 | 211 |
| 23 | 84 | M | 36 | 13.0 | 700 | 100 | 400 | 100 | 183 |
| 24 | 54 | F | 216 | 13.6 | 3300 | 1500 | 1600 | 300 | 263 |
| 25 | 38 | F | 312 | 12.1 | 3300 | 1200 | 1600 | 300 | 241 |
| 26 | 31 | F | 120 | 13.7 | 3600 | 1700 | 1300 | 500 | 210 |
| 27 | 35 | M | 120 | 13.6 | 4500 | 700 | 2100 | 1200 | 320 |
| 28 | 55 | F | 120 | 14.7 | 4100 | 1400 | 2000 | 400 | 195 |
| 29 | 29 | M | 132 | 13.8 | 3000 | 400 | 1800 | 600 | 212 |
| 30 | 31 | F | 84 | 12.7 | 2300 | 300 | 1200 | 600 | 311 |
| 31 | 34 | M | 84 | 15.1 | 2700 | 800 | 1500 | 400 | 197 |
| 32 | 31 | F | 84 | 12.7 | 2300 | 300 | 1200 | 600 | 311 |
| 33 | 67 | M | 50 | 12.1 | 2000 | 600 | 1200 | 200 | 232 |
| 34 | 39 | F | 24 | 13.8 | 4600 | 1600 | 2300 | 400 | 225 |
| 35 | 62 | F | 48 | 14.6 | 5300 | 100 | 4100 | 1100 | 202 |
| 36 | 21 | F | 48 | 13.4 | 3600 | 1400 | 2100 | 100 | 211 |
| 37 | 24 | F | 48 | 11.8 | 3700 | 600 | 2400 | 400 | 288 |
| 38 | 18 | M | 48 | 14.6 | 2000 | 200 | 1300 | 400 | 175 |
| 39 | 79 | F | 240 | 14.4 | 3300 | 1600 | 1400 | 300 | 261 |
| 40 | 29 | F | 36 | 14.5 | 3400 | 900 | 1700 | 500 | 225 |
| 41 | 18 | M | 96 | 14.4 | 3300 | 1000 | 2000 | 300 | 290 |
| 42 | 75 | F | 96 | 12.1 | 2600 | 1100 | 1100 | 300 | 193 |
| 43 | 60 | F | 180 | 12.1 | 3400 | 1500 | 1300 | 400 | 329 |
| 44 | 62 | F | 180 | 12.8 | 4100 | 1700 | 2000 | 300 | 228 |
| 45 | 30 | F | 180 | 12.8 | 3700 | 1400 | 1800 | 300 | 200 |
| 46 | 39 | F | 180 | 12.2 | 2700 | 1400 | 800 | 400 | 258 |
| 47 | 51 | F | 132 | 13.3 | 3400 | 1700 | 1300 | 300 | 295 |
| 48 | 27 | F | 204 | 12.1 | 2900 | 1700 | 1000 | 200 | 267 |
| 49 | 34 | F | 204 | 13.6 | 4000 | 1600 | 2100 | 300 | 190 |
| 50 | 17 | M | 60 | 14.2 | 2200 | 700 | 1100 | 400 | 239 |
| 51 | 52 | F | 120 | 12.2 | 4200 | 1600 | 2300 | 200 | 278 |
| 52 | 52 | F | 180 | 12.2 | 4200 | 1600 | 2300 | 200 | 278 |
| 53 | 29 | M | 60 | 15.1 | 3700 | 600 | 1700 | 1100 | 239 |
| 54 | 43 | F | 60 | 13.9 | 3700 | 1700 | 1400 | 400 | 225 |
| 55 | 67 | M | 60 | 12.4 | 2600 | 700 | 1200 | 600 | 345 |
| 56 | 67 | F | 60 | 13.8 | 3900 | 1700 | 1800 | 300 | 236 |
| 57 | 65 | F | 60 | 13.8 | 4300 | 1700 | 1900 | 500 | 218 |
| 58 | 65 | F | 180 | 13.8 | 4300 | 1700 | 1900 | 500 | 218 |
| 59 | 76 | F | 288 | 12.6 | 3800 | 1500 | 1900 | 300 | 211 |
| 60 | 53 | F | 252 | 12.2 | 4200 | 1400 | 2600 | 200 | 241 |
| 61 | 32 | M | 132 | 12.8 | 3300 | 1200 | 1700 | 300 | 190 |
| 62 | 63 | F | 252 | 13.1 | 4200 | 1600 | 1900 | 500 | 219 |
| 63 | 45 | F | 132 | 14.0 | 3500 | 1600 | 1600 | 200 | 204 |
| 64 | 70 | F | 252 | 12.2 | 4700 | 1000 | 3100 | 400 | 215 |
| 65 | 30 | M | 132 | 13.8 | 3000 | 400 | 1800 | 600 | 212 |
| 66 | 51 | F | 252 | 12.2 | 2700 | 1000 | 1400 | 200 | 227 |
| 67 | 51 | F | 132 | 12.2 | 2700 | 1000 | 1400 | 200 | 227 |
| 68 | 23 | M | 60 | 14.2 | 3500 | 1700 | 1400 | 300 | 272 |
| 69 | 70 | F | 300 | 13.1 | 4200 | 1600 | 1900 | 500 | 219 |
| 70 | 42 | M | 144 | 13.8 | 2400 | 300 | 1200 | 700 | 150 |

**Abbreviations:** CIN, chronic idiopathic neutropenia; UPN, unique patient number; Hb, hemoglobin; WBC, white blood cells; Neutro, neutrophils; Lympho, lymphocytes; Mono, monocytes; Plts, platelets.
